# Transcriptional interference networks coordinate the expression of functionally related genes clustered in the same genomic loci

**DOI:** 10.3389/fgene.2012.00122

**Published:** 2012-07-05

**Authors:** Zsolt Boldogköi

**Affiliations:** Department of Medical Biology, Faculty of Medicine, University of Szeged,Szeged, Hungary

**Keywords:** transcriptional interference, antisense RNA, polycistronic RNAs, tandem genes, genomic organization, herpesvirus, pseudorabies virus, *Hox* genes

## Abstract

The regulation of gene expression is essential for normal functioning of biological systems in every form of life. Gene expression is primarily controlled at the level of transcription, especially at the phase of initiation. Non-coding RNAs are one of the major players at every level of genetic regulation, including the control of chromatin organization, transcription, various post-transcriptional processes, and translation. In this study, the Transcriptional Interference Network (TIN) hypothesis was put forward in an attempt to explain the global expression of antisense RNAs and the overall occurrence of tandem gene clusters in the genomes of various biological systems ranging from viruses to mammalian cells. The TIN hypothesis suggests the existence of a novel layer of genetic regulation, based on the interactions between the transcriptional machineries of neighboring genes at their overlapping regions, which are assumed to play a fundamental role in coordinating gene expression within a cluster of functionally linked genes. It is claimed that the transcriptional overlaps between adjacent genes are much more widespread in genomes than is thought today. The Waterfall model of the TIN hypothesis postulates a unidirectional effect of upstream genes on the transcription of downstream genes within a cluster of tandemly arrayed genes, while the Seesaw model proposes a mutual interdependence of gene expression between the oppositely oriented genes. The TIN represents an auto-regulatory system with an exquisitely timed and highly synchronized cascade of gene expression in functionally linked genes located in close physical proximity to each other. In this study, we focused on herpesviruses. The reason for this lies in the compressed nature of viral genes, which allows a tight regulation and an easier investigation of the transcriptional interactions between genes. However, I believe that the same or similar principles can be applied to cellular organisms too.

## INTRODUCTION

### THE RATIONALE BEHIND THE OPERATION OF TINs

The TIN hypothesis assumes that the process of transcription itself plays a regulatory role in the coordination of gene expression within a cluster of functionally linked genes. The mechanism of this coordination is based on the confrontation of the transcriptional machineries at the various overlaps formed by neighboring genes of a genetic modules (GM). The rationale behind the operation of the TINs lies in providing a straightforward genetic algorithm to coordinate the ON/OFF transcription pattern of functionally linked gene clusters, which would otherwise be possible only via sophisticated mechanisms, including continuous monitoring of the actual state of gene expression and modification of the transcription profiles of the genes accordingly. In a cluster of tandem genes, transcription of an upstream gene causes the inhibition of the downstream genes. This mechanism represents a unidirectional interaction between these genes (Waterfall model). On the contrary, the various convergent overlaps allow a two-way interplay between adjacent genes, resulting in a mutually exclusive expression of the interacting partners (Seesaw model). Besides the collision-based models, another way of interference between genes occurs through the competition between the transcriptional machineries for determining the direction of the transcription from bidirectional promoters. The collision of the transcriptional apparatuses results in a non-linear effect in gene expression. The outcome of the collisions is dependent on two important factors, which are the activity of the promoter and the efficiency of the read-through. Both processes are differentially regulated and can change in time. The outcome of the interaction between two convergent genes is controlled by the frequency of transcriptional read-through; the genes with higher rate will win. Genes with initially higher activity and/or higher transcriptional read-through efficiency can significantly or entirely suppress the expression of genes with lower initial activity (the “Winner takes all” principle), for which the reason is at least threefold. First, the initiation step of transcription is a time-consuming process, which can therefore be effectively blocked via the rapidly advancing RNA polymerase II (RNAP) from a gene with high transcriptional activity. Second, the clash of the RNAP molecules occurs more frequently at the loci (half-space) of genes with lower activity in the case of convergent read-through overlaps. The reason for the unequal effect of the collisions is that the dislocation of the RNAP molecule during the transcription of a coding region results in the generation of functionless transcripts, but does not significantly influence translation in the read-through part of the transcripts. Furthermore, clashing of RNAPs outside the gene locus enables the production of a large population of normally terminated mRNA molecules (non-read-through transcripts). According to the Seesaw model, transcriptional collisions produce a self-regulatory automatism whereby the higher-rate transcription mechanically makes gene expression topple over from one kinetic (sub)class to another, subsequently maintaining the switched state for a given period of time. In other words, the expression of a gene is not only dependent on the presence or absence of transcription factors and the epigenetic state of the given locus, but also on the activity status of adjacent genes and the control of poly(A) recognition by factors such as ICP27 in herpesviruses (McLauchlan et al., 1992; McGregor et al., 1996). The TINs provide an auto-regulatory mechanism that controls the stepwise turning on and off of genes in a controlled manner. Furthermore, the operation of the TINs results in inverse synchronization, fine tuning, and economizing of gene expressions of adjacent genes.

### GENETIC REGULATION

The recent view held that transcription is controlled by the combinatorial action of transcription factors and other regulatory proteins through binding to the enhancer and promoter sequences of the genes. This notion had its roots in the biochemical perspective that proteins comprise the structural and core catalytic framework of the cell. RNAs were thought to play subsidiary roles compared to the two truly important molecules: DNA, which carries the genetic information, and proteins, which execute nearly every function in the cell. However, this picture has been dramatically changing in recent years with the realization that RNAs have far more important functions in cells than was considered before (Weinberg et al., 2009). Alpha-herpesviruses have relatively large double-stranded DNA genomes containing more than 70 genes. These viruses have a very compact genomic structure with few introns and very short intergenic regions; therefore, they serve as ideal models to investigate eukaryotic transcription regulation. An additional beneficial feature of these viruses is that their replication cycle is rapid, requiring less than 24 h to produce infectious progeny. The program of herpesvirus gene expression is primarily controlled at the level of transcription. The lytic cycle of the herpesviruses can be characterized by the tightly coordinated expression of viral proteins belonging to different temporal groups, which are traditionally termed immediate-early (IE), early (E), early/late (E/L), and late (L) kinetic classes. The IE proteins are transcription factors that control the transcription of other viral genes. Most of the E proteins are required for the synthesis of viral DNA, while the L proteins mainly serve structural functions making up the capsid, tegument, and envelope, as well as promoting virion maturation (Roizman, 1996; Boldogköi and Nógrádi, 2003; Pomeranz et al., 2005). Besides the lytic pathway, herpesviruses can also establish a life-long latent infection in various cell types. The *Hox* genes are a set of transcription factor genes that are instrumental in regulating the timing and route of animal body formation during development. They belong to the homeotic gene family, which is characterized by a DNA sequence of approximately 180 base pairs called the homeobox encoding the homeodomain, a DNA-binding segment of proteins. *Hox* proteins act like genetic switches, turning off and on other genes with a mechanism whose key parameter is the gene dosage. The *Hox* genes themselves are regulated by more posteriorly acting *Hox* proteins and several other types of transcription factors. Many *Hox* genes have remarkably conserved functions. For example, the *Pax6* gene is involved in eye development both in insects and vertebrates, and is even expressed in the photoreceptor cell of the *Cnidarian* species. Vertebrate *Hox* genes are organized into four clusters (A, B, C, and D), while invertebrates contain only a single set of *Hox* genes. One of the most striking properties of the *Hox* genes is that they are activated in a collinear fashion; that is, the relative order of their expression along the anterio-posterior axis correlates with the position of the genes in the gene cluster.

### GENOME ORGANIZATION

**FIGURE 1:**
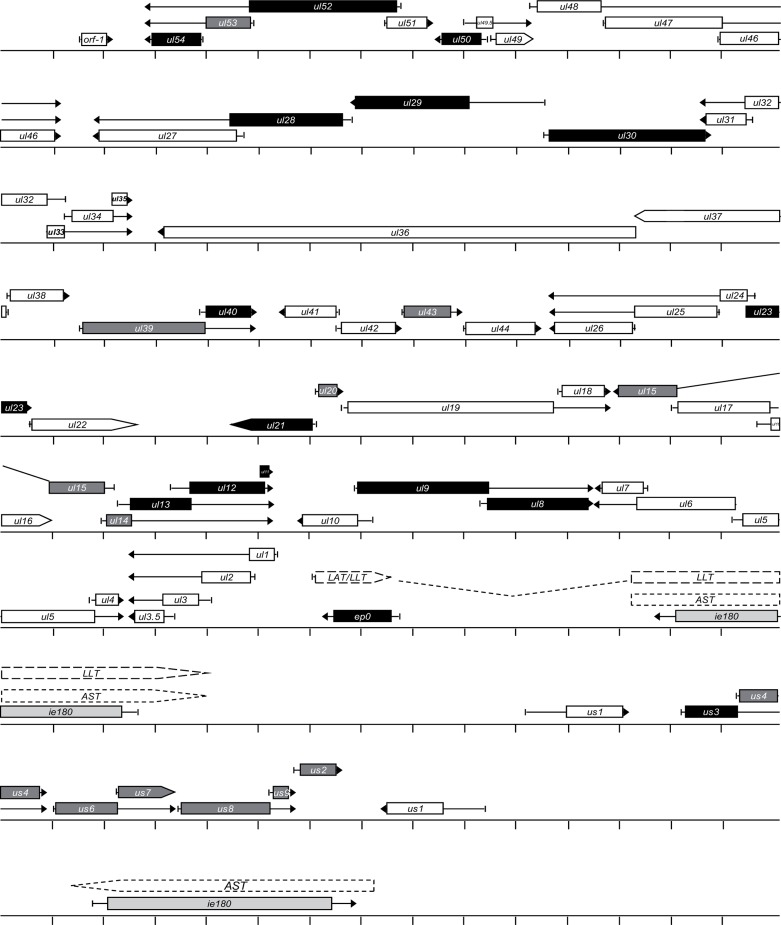
**The structural organization of the pseudorabies virus genome**. The genome of PRV is atypical among alpha-herpesviruses because it does not have inverted repeat sequences around the Ul region. Color code: light grey, immediate early gene (i.e., 180 gene); black, early genes; dark grey, E/L genes; white, L genes.

The genes of bacteria are organized into groups of transcriptionally linked genes called operons, whereas eukaryotic genes have been traditionally thought of as having non-operon-like structures. In fact, the genes of complex eukaryotes are non-randomly distributed in the DNA; they form small “islands” on the chromosome, which are surrounded by large “deserts” of non-coding DNA. It has been demonstrated that genes residing at the same genomic locus tend to be co-expressed (Lercher et al., 2002). In an attempt to explain this phenomenon, several hypotheses have been put forward, mainly on the basis of the assumption of selection for the co-regulation of functionally related genes (Lee and Sonnhammer, 2003; Peri et al., 2003; Hurst et al., 2004), but neutral components of the co-expression are also emphasized (Michalak, 2008). The co-localized genes have evolved either from a shared ancestor by gene duplication or recruited from distinct loci of the same chromosome or from different chromosomes. It has been estimated that around 38% of the human genes and 49% of the *Caenorhabditis elegans* genes arose from gene duplication (Rubin et al., 2000; Li et al., 2001). The actual proportions may be even higher, since ancient duplications cannot be recognized through a homology search on genome databases. Tandemly arrayed genes (TAGs) are a result of tandem duplications on the chromosomes (they can be separated by spacer genes, which are not homologous to the members of TAGs). A survey of 11 vertebrate genomes revealed that TAGs account for some 14% of all genes in these genomes (Pan and Zhang, 2008). It has been demonstrated that most of the tandem arrays (60–83% in the various species) are composed of two members, and that the majority of duplicated genes (72–94%) exhibit a parallel transcription orientation (Yi et al., 2007). By using *in silico* analysis, Al-Shahrour et al. (2010) assembled a detailed functional cartography for the genomes of eight eukaryotic model species (human, chimpanzee, mouse, rat, chicken, zebra fish, fruit fly, *C. elegans*, and *Arabidopsis thaliana*), and revealed that a large fraction of the DNA is arranged in neighborhoods of functionally related genes. Intriguingly, they further found that these functional gene clusters were generated more frequently through the reorganization of the genome than through gene duplication. Genes of a cluster may form interaction networks, in which the proteins interact with each other directly by forming multimeric proteins, or serving as upstream and downstream components in the signaling pathways (Hurst et al., 2004; Teichmann and Veitia, 2004), or through the mutual regulation of their expressions, forming gene networks of transcription factor genes (e.g., *Hox* genes). Alternatively, co-localized genes can feature in the same biochemical pathway without their protein products participating in direct interactions, e.g., enzymes taking part in a common metabolic pathway, β-globins carrying oxygen in the muscle, or in fetal or adult blood, or tRNAs and MHC proteins involved in the translation and immune defense, respectively. In either case, the clustering of genes appears to confer a selective advantage for the formation and maintenance of gene clusters. Besides the coordinated gene expression, the selective pressure may also favor the long-term linkage (co-inheritance) of clustered genes. For example, the existence of large regions of linkage disequilibrium has been reported among inbred mouse lines, which were shown to be correlated with the biological function (Petkov et al., 2005). Additionally, it has been reported that clusters of homologous genes in *C. elegans* tend to comprise species-specific gene families that play roles in detoxification and immunity (Thomas, 2006). In the genome of pseudorabies virus (PRV), the functionally linked genes appear to be clustered: the genes *rr1* and *rr2* are involved in the synthesis of viral DNA (Kaliman et al., 1994), the genes *ul18–21* in capsid maturation (see references in Tombácz et al., 2009), etc. (**Figure 1**). However, the adaptive origin and maintenance of genetic networks in complex eukaryotes has been called into question in several publications (e.g., Lynch, 2007a). It is claimed that the “strong” effect of natural selection and a “weak” effect of genetic drift in unicellular organisms and viruses could have led to the emergence of functional gene clusters for the coordinate regulation of metabolic genes in prokaryotes and unicellular eukaryotes (Lynch and Conery, 2003; DeLong et al., 2010), and of replication and host recognition genes in large DNA viruses (e.g., Shackelton and Holmes, 2004). However, the evidence appears to suggest that the effect of genetic drift in multicellular eukaryotes is more considerable than that of natural selection, which compromises the formation and maintenance of functional gene clusters and their interactions in regulatory networks (Lynch, 2007a,b). Further data are needed to clarify this issue.

**FIGURE 2:**
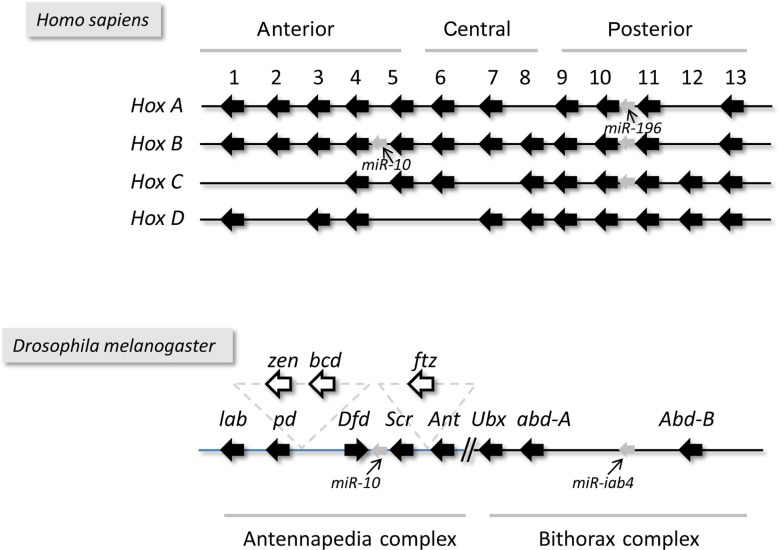
**The structural organization of the *Hox* clusters**. *Drosophila* has a single set of *Hox* genes termed the Homeotic complex, which is made up of two clusters, the *Antennapedia* and the *Bithorax* complexes. Vertebrates have four sets of *Hox* clusters generated by two rounds of polyploidization events that occurred prior to the divergence of jawless and jawed vertebrates.

In sum, the tandem arrangement of functionally related genes on the DNA appears to represent a common organization principle in both cellular organisms and viruses with large genome sizes. Mammalian *Hox* genes are all arrayed in a tandem orientation, while the arrangement of some *Hox* genes in arthropods (insects and crustaceans) has been reversed during evolution (**Figure 2**). In various arthropod species, genomic reorganization events have generated antiparallel gene pairs within the *Hox* gene cluster (**Figure 2**). Herpesviral genes exhibit a characteristic modular organization, which includes two convergently oriented gene clusters, each containing parallelly overlapping genes (**Figure 1**). Questions arise as to whether this modular genomic organization is common in cellular organisms too and if so, whether it has any functional significance or represents a mere curiosity without any apparent function.

### NON-CODING RNAs

#### Genome-Wide transcription Of non-coding RNAs

Current technological advances have allowed the investigation of overall expression of human and mouse genomes with an exceptional resolution. These analyses have revealed that the major parts of these genomes are transcribed and that protein-coding sequences account for only a small proportion of the total transcriptional output (Bertone et al., 2004; Cheng et al., 2005; Birney et al., 2007; Kapranov et al., 2007). There is still ongoing debate as to whether this pervasive transcription represents anomalies for the most part or if the non-coding RNAs (ncRNAs) have functions that have not yet been identified (Mattick, 2004; Struhl, 2007; Ebisuya et al., 2008; Kapranov and St. Laurent, 2012). The transcriptional noise hypothesis implies that cells can benefit from allowing some random transcription to occur rather than suppressing non-specific transcription, and this appears to be supported by the fact that individual ncRNAs are transcribed at much lower levels than individual mRNAs (Louro et al., 2009). In the past couple of years, multiple types of these “dark matter RNAs” have been discovered with diverse functionality. This unforeseen level of transcriptome complexity has led to the realization that ncRNAs comprise a formerly hidden layer of genetic programming in multicellular organisms. NcRNAs appear to form complex regulatory circuits, which control gene expression at multiple levels (Mattick, 2010).

#### Natural antisense transcripts

Natural antisense transcripts (NATs) represent a group of ncRNAs that have complementarity to other transcripts. Many loci of chromosomes contain transcription units on both DNA strands. Often, one transcript encodes a protein, whereas the RNA from the complementary DNA strand is non-coding. These latter RNA molecules are termed *cis*-NATs. In contrast, *trans*-NATs are encoded outside the genomic loci that specify the target mRNAs. *Cis*-NATs are entirely complementary to their mRNA partners, whereas *trans*-NATs generally display imperfect complementarity to their target mRNAs (Li et al., 2006). NATs can occur in promoters, enhancers, introns, exons, intergenic sequences, and untranslated regions of the genome (Li and Ramchandran, 2010). NATs have been shown to exert control on their target genes at various levels, including transcription, RNA processing, splicing, stability and cellular transport of RNAs, and translation. The most prominent representatives of *trans*-NATs are the microRNAs (miRNAs) and the endogenous small interfering RNAs (siRNAs), although these transcripts can be encoded in *cis*, too. MiRNAs comprise short non-coding transcripts, which are post-transcriptional regulators that bind to complementary sequences on target mRNAs and modulate gene activity by interfering with mRNA stability or translation (Carthew, 2006; Bartel, 2009). Small RNAs can repress transcription in two alternative ways: either through chromatin modification (DNA methylation or histone remodeling) or, alternatively, through targeting the transcription start sites, thereby blocking procession and/or recruiting of RNAP (Janowski et al., 2005; Napoli et al., 2009). The human genome encodes over 1,000 different miRNAs, which are supposed to target at least 60% of the mammalian genes (Friedman et al., 2009).

#### Long non-coding transcripts

Recent attention has focused on miRNAs. However, the most abundant and least annotated class of ncRNAs is the long non-coding RNAs (lnc-RNAs; Mattick and Makunin, 2006), which are defined as transcripts exceeding 100 nucleotides without containing functional open reading frames (ORFs). The mouse transcriptome was recently calculated to contain approximately 180,000 transcripts; however, only 20,000 of them encode proteins (Carninci et al., 2005). It has been shown that a large proportion of the human DNA also encodes lnc-RNAs (Wilusz et al., 2009). Many protein-encoding genes specify lnc-RNAs transcribed from the sense (plus) DNA strands as templates, which are called antisense lnc-RNAs. Long overlapping transcripts can include two protein-encoding mRNAs, an mRNA/lnc-RNA pair or two lnc-RNAs. Transcriptome analysis of antisense lnc-RNAs and their mRNA partners revealed frequent concordant regulation of expression (Katayama et al., 2005). Furthermore, it has been shown that sense-antisense pairs often exhibit reciprocal expression patterns (Lehner et al., 2002), indicating interplay between these two RNA molecules. The antisense lnc-RNAs have been reported to suppress the expression of their mRNA counterparts in cultured human cells (Izant and Weintraub, 1985). While several reasonable hypotheses have been put forward in an attempt to explain how the antisense lnc-RNAs regulate mRNA expression, there is little experimental evidence supporting these explanations (Stolt and Zillig, 1993; Rinn et al., 2007; Roeszler et al., 2012). Data generated over the past few years have provided insights into these mechanisms, which involve, among others, remodeling of the chromatin structure at target loci by antisense lnc-RNAs (Morris and Vogt, 2010) and transcriptional interference (TI) between the transcriptional machineries at the overlapping DNA region (Martens et al., 2004).

#### Transcription from bidirectional promoters

Current sequencing and annotation methods have revealed that divergent organization of coding genes is widespread in various genomes (Trinklein et al., 2004; Hermsen et al., 2008). It has also been pointed out that eukaryotic promoters are intrinsically bidirectional (Wei et al., 2011). The function of bidirectional promoters has long been held to provide joint coordination of expression of two divergent genes, whose protein products are typically involved in the same biochemical function (Meier and Straus, 1993). However, it has recently been demonstrated that bidirectional promoters, are involved in the generation of ncRNAs called cryptic unstable transcripts (CUTs; Neil et al., 2009). CUTs have been described in various eukaryotic species, ranging from yeasts (Xu et al., 2009) to mammals (Core et al., 2008; Seila et al., 2008). There have been speculations on the potential function of CUTs. It is possible that the process of CUT transcription results in the nucleosomes being repelled from the promoters, thereby allowing either prolonged access of the transcription factors to the *cis*-regulatory elements (Mavrich et al., 2008), or the linking of the promoter region with the 3′-end of the gene (Tan-Wong et al., 2009). Alternatively, the CUTs themselves could be functional, through RNA interference (Han et al., 2007) or Argonaute-independent (Wang et al., 2008) silencing pathways.

#### Antisense RNA expression in herpesviruses

MiRNAs have been shown to be encoded in all of the three subfamilies of herpesviruses, including herpes simplex virus (HSV; alpha-herpesvirus, Umbach et al., 2008), human cytomegalovirus (beta-herpesvirus; Grey et al., 2005), and Epstein–Barr virus (gamma-herpesvirus; Pfeffer et al., 2004). Although the precise functions of most viral miRNAs remain to be ascertained, an increasing number of their viral and cellular targets have been experimentally confirmed. Herpesviral miRNAs are supposed to play a pivotal role in the latent/lytic switch during the viral life cycle, the evasion of host immune responses, and preventing apoptosis of the host cell (Li et al., 2010). Numerous long *cis*-antisense transcripts have been identified in human cytomegalovirus (Zhang et al., 2007). The latency associated transcript (LAT) of HSV was described as the first viral *cis*-NAT (Stroop et al., 1984). The LAT region encodes multiple transcripts, including the 8.3-kb primary transcript and two stable introns of 1.5 and 2.0 kb (Zwaagstra et al., 1990; Farrell et al., 1991). The LAT plays a key role in establishing and maintaining viral latency (Perng et al., 2000). A spliced 8.4-kb antisense RNA, termed long latency transcript (LLT), has been shown to be synthesized from the complementary DNA strand of *ie180* and *ep0* genes driven by the LAT promoter of PRV, a veterinary alpha-herpesvirus (Cheung, 1989; Priola and Stevens, 1991). The antisense transcript (AST) RNA gene overlapping the *ie180* gene has been suggested to be under the control of a separate promoter, termed antisense promoter (ASP; Boldogköi et al., 2000; **Figure 4**). Antisense RNA expression has also been reported in some other HSV genes (Ward et al., 1996; Chang et al., 1998; Jovasevic and Roizman, 2010). Furthermore, we have detected an overall antisense RNA transcription in PRV using real-time RT-PCR techniques (unpublished result). Together, antisense transcripts appear to be a pervasive feature of herpesviruses, suggesting that they are fundamental components for the regulation of the viral life cycle.

#### Antisense RNAs in the Hox gene clusters

Mammalian *Hox* clusters have been shown to contain five miRNA genes intercalated at two homologous positions (Kosman et al., 2004). Furthermore, Mainguy et al. (2007) reported the existence of *cis*-encoded antisense RNAs in the *Hox* gene cluster. Antisense transcripts were found to represent approximately 38% of the total spliced transcripts. It has been shown that *HOTAIR*, an antisense lnc-RNA transcribed from the *HOXC* locus, affects gene expression in the *HOXD* locus residing on a different chromosome (Rinn et al., 2007). Indeed, targeting *HOTAIR* transcripts by RNA interference results in the transcriptional activation of the *Hox*D locus (Rinn et al., 2007), indicating an inhibitory role of these transcripts. *HOTAIR* has been shown to be a regulator of the chromatin state by providing binding surfaces for the assembly of histone modification enzymes (Tsai et al., 2010).

### Polycistronic RNAs

Most of the mRNAs found in eubacteria and archaea is polycistronic, and are transcribed from the structural genes of an operon. These RNAs contain ribosome-binding sites at an upstream location of each gene. On the other hand, eukaryotic mRNAs have only a single site for the initiation of protein synthesis; therefore, with a few exception (e.g., *Hox* genes), they are thought to be monocistronic. *Hox* genes are tandemly arranged in an order collinear with their expression along the anterior–posterior axis. Extensive polycistronism has been reported in mammalian *Hox* gene clusters, where *Hoxc6*, *Hoxc5*, and *Hoxc4* are co-transcribed (Simeone et al., 1988). The mammalian *Hox* clusters are estimated to display at least seven polycistronic clusters (Mainguy et al., 2007). Notably, polycistronic *Hox* transcripts have also been described in a number of crustacean species (Shiga et al., 2006), indicating their importance in diverse evolutionary lineages. Several downstream *Hox* genes on polycistronic mRNAs have been reported to be translated by means of internal ribosomal entry (Oh et al., 1992) or alternative splicing (Simeone et al., 1988; Mainguy et al., 2007). Tandem herpesviral genes are embedded in each other in such a way that the transcription from the upstream genes continues across the transcription termination signals with certain efficiencies. This process is regulated by the ICP27 viral protein, which helps recognize the weak poly(A) signals by RNAP (McGregor et al., 1996); that is, in the absence of ICP27, the parallelly embedded genes will produce long, polycistronic mRNAs with common 3′-termini. Many viral species solved the problem of translating multiple messages from a polycistronic mRNA by developing various mechanisms such as: alternative splicing of primary RNA products in HIV (Schwartz et al., 1990); leaky scanning of *vpu* and *env* transcripts in HIV, as well as the OFR1 and ORF2 genes in rotavirus; ribosomal frame shifting of *gag* and *gag-pol* genes in HIV; and the utilization of internal ribosomal entry sites (IRES) in picornaviruses and hepatitis C virus (reviewed by Stacey et al., 2000). However, none of the above mechanisms has been described in herpesviruses so far. Thus, it is presently unknown as to whether downstream ORFs of polycistronic RNAs are translated in these viruses (Sokolowski et al., 2003).

### INTERFERENCE BETWEEN THE TRANSCRIPTIONAL MACHINERIES

Transcription regulation in eukaryotic cells is thought to be controlled by transcription factors through binding to the *cis*-regulatory response elements, thereby promoting or inhibiting the efficiency of the pre-initiation complex assembly or the activity of RNAP. Reports have been recently published on a different regulatory mechanism based on the interference between the transcriptional machineries of adjacent genes. The phenomenon of transcriptional collision of RNAP molecules in oppositely oriented genes has been demonstrated in yeast (RHO1 and MRP2 genes, Peterson and Myers, 1993; GAL7, GAL10, and TI genes, Prescott and Proudfoot, 2002). These convergent yeast gene pairs overlap, that is, their transcription termination sequences are embedded within each other’s transcribed DNA regions. In these examples, the elongation phase of transcription of both genes was found to be affected, while the transcription initiation was unaltered. In another publication, transcriptional collisions were reported between RNAPs transcribing mRNAs and *cis*-NATs in several murine and human genes (Osato et al., 2007). However, no promoters were identified experimentally or *in silico*, which would control the expression of *cis*-NATs. A similar mechanism was also described in yeasts: lnc-RNA molecules transcribed from a non-protein encoding genomic region were shown to directly interfere with the binding of transcription factors to the promoter of adjacent SER3 gene, thereby preventing this gene from being expressed (Martens et al., 2004). The consequence of the collision of RNAP molecules has not yet been investigated. A theoretical possibility is that one or both RNAPs are released from the template. Alternatively, confronted RNAP molecules may stall and be unable to advance further. Any of the above mechanisms would severely impede the transcription of both genes. TI between the mRNAs and antisense lnc-RNAs has been shown to regulate key developmental decisions, such as where *Hox* genes are expressed (Petruk et al., 2006). Bithoraxoid (*Bxd*) is an lnc-RNA found in *Drosophila*, which silences the expression of the Ultrabithorax (*Ubx*) gene through TI. Furthermore, the amount of transcripts synthesized from tandem genes in the yeasts genome decreases when the transcriptional termination sequences of the upstream gene are removed (Osato et al., 2007), indicating a negative effect of the transcriptional read-through on gene expression. Interaction between the transcriptional apparatuses has not been described in herpesviruses to date.

## HYPOTHESIS AND DISCUSSION

### THE TRANSCRIPTIONAL INTERFERENCE NETWORK HYPOTHESIS

**FIGURE 3:**
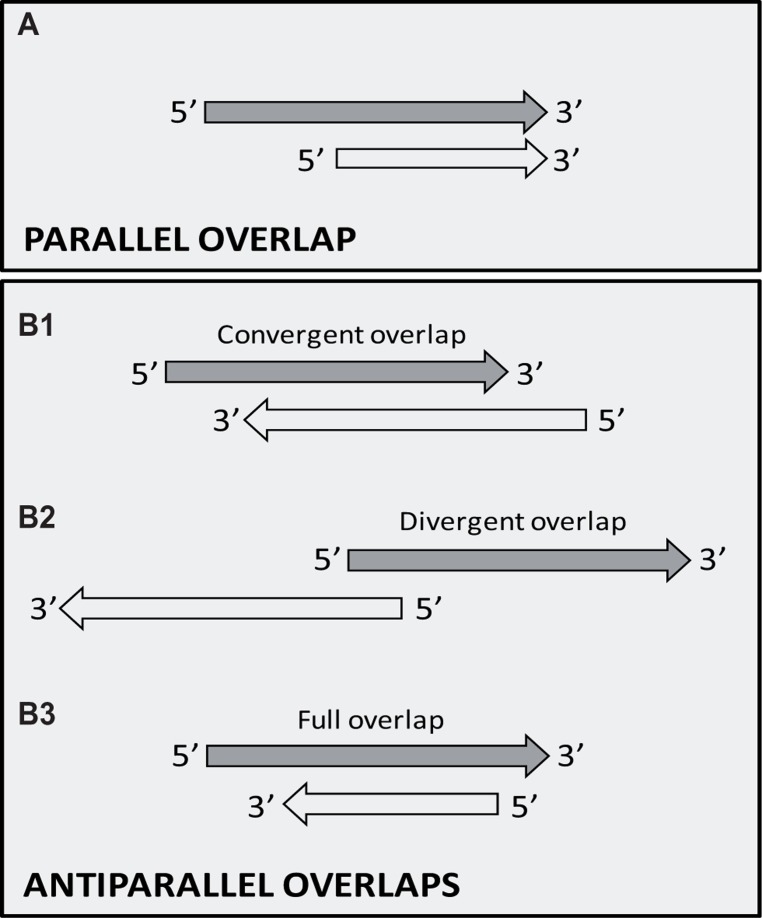
**Possible overlaps producing antiparallel transcripts**. **(A)** Tandem (parallel, head-to-tail, 5′–3′) overlap. Transcription of the upstream genes of a tandem gene cluster often fails to stop at their poly(A) signal, thereby producing long polycistronic RNA molecules. **(B)** Convergent (antiparallel) overlaps. (**B1**) Head-to-head (5′–5′) overlap between two divergently positioned genes. (**B2**) Tail-to-tail (3′–3′) overlap between convergently oriented genes. Two opposing genes can overlap at their 3′-UTR regions (a) or can exhibit a 3′-UTR–ORF (b), or ORF–ORF overlap. (**B3**) Transcriptional read-through overlap occurs when the major poly(A) signal occasionally fails to terminate by the advancing RNAP (a). The use of alternative polyadenylation provides a mechanism similar to that of transcriptional read-through for the spatiotemporal control of gene expression (b).

The TIN hypothesis has been put forward in an attempt to provide a conceptual framework for understanding the potential role of the widespread occurrence of gene clusters with tandemly arrayed members, and the overall antisense RNA expression in biological systems belonging to various taxonomic groups. The TIN hypothesis asserts that genes are organized into structural and functional units, termed GMs, which are comprised of tandemly or convergently oriented genes, or more typically, combinations of them. The hypothesis suggests that the transcription of neighboring genes overlaps in different ways (head-to-tail, head-to-head, tail-to-tail, or full overlap; **Figure 3**), resulting in the confrontation of their transcriptional machineries at the overlapping region. Furthermore, it is proposed that this form of genetic regulation occurs at a genome-wide scale and, therefore, plays an essential role in controlling global gene expression in both cellular organisms and viruses. Convergently overlapping genes can be protein-coding genes or RNA genes, or the combination of them. True convergently overlapping protein-coding genes, especially those with ORF overlaps, are rare in various genomes. However, the TIN hypothesis proposes that in many gene pairs, transcription from convergently positioned genes is not terminated with perfect efficiency at their poly(A) sequences. Instead, transcription occasionally continues at the locus of the opposing gene, which leads to the generation of long RNA molecules containing mRNA sequences from the given gene and antisense RNA sequences from the oppositely oriented gene. The process of alternative polyadenylation can also lead to differential transcriptional overlaps. The long transcripts, generated by the read-through mechanism, appear to be the mere by-products of this process; however, it cannot be excluded that they are further utilized at other levels of genetic regulation. Likewise, downstream-positioned genes of polycistronic RNAs, if untranslated, are also superfluous by-products of the transcriptional read-through between tandemly oriented genes. The potential use of these polycistronic RNAs, if at all, is unknown. Another way of TI is based on the competition of transcription apparatuses on the divergent promoters to determine the direction of transcription. According to the TIN hypothesis, transcription of neighboring genes can depend on each other in either a one-way (parallelly overlapping genes) or two-way direction (convergently overlapping genes). The interaction between the transcriptional machineries provides a mechanism for the coordinate control of the expression of functionally linked genes using a simple genetic algorithm: “keep a gene silent while the adjacent gene is transcribed through the process of transcription itself,” which would be a very complicated task to accomplish in a large gene cluster by means of transcription factors alone. A TIN regulates the transcription switch between two or more genes and acts thereafter to maintain the turned-on state of a gene and the turned-off state of the neighboring genes for a given period of time. The interdependence of gene activities provides an in-built automatism for producing a tightly choreographed succession of expression patterns of co-localized genes. To coordinate gene expression at a broader scale, GMs themselves can also interact with each other in various ways, including the TI within the divergent overlaps of upstream genes of adjacent GMs or the transcriptional read-through from distant genes belonging to other GMs. The operation of TINs will now be discussed in the alpha-herpesviruses, especially in the PRV, which is a useful model organism for studying the molecular pathogenesis of herpesviruses (Boldogköi et al., 2004) and is also a beneficial tool in neuroscience (Boldogköi et al., 2009).

#### The Waterfall model

***Tandemly overlapping genes***. Functionally coupled genes are often arrayed in tandem along a DNA segment in various cellular organisms and viruses. Many of these genes have been generated by gene duplication, but the conservation of this architecture over a long evolutionary timescale requires an explanation. In an attempt to explain this phenomenon, Huvet et al. (2007) proposed a hypothesis that was based on the collision of the replication and transcription machineries; however, the experimental verification of this model, at least in human cells, failed (Necsulea et al., 2009). The Waterfall model of the TIN hypothesis provides an alternative, but not necessarily exclusive, explanation for this subgenomic organization, which is based on the TI between tandemly overlapping genes. Accordingly, the upstream-positioned genes exert an inhibitory effect on the expression of downstream genes through their unterminated transcription. In herpesviruses, this process is regulated by the ICP27 protein, which helps recognize the weak transcription termination signals of the RNAP molecules (McGregor et al., 1996). As a result of transcriptional read-through, parallelly oriented genes generate long polycistronic mRNAs with common 3′-termini. Most of the tandem gene clusters of PRV are composed of genes with similar transcription kinetics (Tombácz et al., 2009). We assume, however, that the transcription of these genes is slightly shifted in time relative to each other, which is difficult to verify experimentally, even with highly accurate techniques such as real-time RT-PCR. The reason for this is that downstream genes are located on both monocistronic and polycistronic transcripts, but in the latter case, these genes are very likely untranslated; that is, the “effective” (translated) amount of transcripts is unknown. The transcriptional machineries of tandem genes collide and, as a result, transcription of the upstream genes suppresses the expression of downstream genes by inhibiting the assembly of their transcription pre-initiation complex. Thus, a gene with early expression kinetics can effectively block the transcription of a downstream gene. Following the cease of the blocking effect by the upstream gene (termination of the transcription or the read-through), the downstream gene is transcribed with a delayed kinetic shift. Likewise, an upstream gene with late expression kinetics can terminate the transcription of downstream-positioned early genes. Poly(A) site selection, as a form of post-transcriptional regulation, has been described to be influenced both by the inherent poly(A) site “strength” and by the concentrations of processing factors (Lassman and Milcarek, 1992). Together, the expression properties of a gene are not solely determined via the interaction between its *cis*-regulatory elements (promoters and enhancers) and transcription factors, but also via the activity or silence of upstream genes and the control of the transcriptional read-through efficiency. In the Waterfall model, the downstream genes do not exert any direct effect on the transcription of upstream genes; therefore, this type of interaction results in a one-way dependence of gene expression (here comes the name “waterfall”). Nonetheless, producing polycistronic RNAs can slow down the transcription of upstream genes due to the longer time needed for the production of polycistronic RNAs compared to monocistronic ones. The downstream genes of *Hox* clusters are controlled by the transcription factors encoded by the upstream genes, thereby resulting in a spatiotemporally well-controlled stepwise activation of these genes. According to the Waterfall model, the same pattern of gene expression can be achieved through the operation of TINs.

#### The Seesaw model

The Seesaw model of the TIN hypothesis proposes a mutually interdependent mode for regulating the expression of neighboring genes. The seesaw mechanism is based on the head-on collision of RNAP molecules during the transcription of two or more overlapping genes positioned in convergent or divergent orientations relative to each other.

***Divergently overlapping genes***. Divergently oriented genes can often overlap and generate transcripts with 5′ overlapping regions (Wei et al., 2011). All of the 11 divergent gene pairs of PRV transcriptionally overlap in the following manners: 5′–5′-UTR (untranslated region) overlap in the *ul41*–*ul42* gene pair; 5′-UTR–ORF overlap in the *ul37*–*ul38* gene pair; and ORF–ORF overlaps in seven PRV gene pairs, intriguingly, four of them overlap with two nucleotides at the AUG translation initiation codons. Furthermore, 2 of the 11 divergent PRV genes have a common TATA-box (see in Gu et al., 2009). The Seesaw model predicts that the transcription of one gene inhibits the transcription of the divergently overlapping partner by preventing the assembly of its transcription initiation complex and/or dislocating its RNAP molecules from the DNA template. At a later phase of viral replication, the direction of transcription alters due to the formation of a new combination of transcription factors and/or the activation or repression of these factors. Therefore, the gene, which was earlier suppressed, is transcribed, which then results in the inhibition of the transcription of the opposing gene (hence, the name “seesaw”). This mechanism operates according to the “Winner takes all” principle, where the “winner” is the gene with the higher initial rate of expression. Our analysis revealed that four of the nine divergent overlaps occur between the L genes (*ul5*–*ul6*, *ul32*–*ul33*, *ul37*–*ul38*, and *ul41*–*ul42*), one between the E/L genes (*ul14*–*ul15*), and four between an E and an L gene (*ul9*–*ul10*, *ul23*–*ul24*, *ul49.5*–*ul50*, and *ul51*–*ul52*), while there is no overlap between the E genes (Tombácz et al., 2009). Although overlapping of divergent genes contributes to genome compaction by eliminating DNA sequences between genes, we believe that its function also includes a TI-based regulation of gene expression. The Seesaw model predicts that divergently overlapping genes all belong to different kinetic classes or subclasses using a finer scale categorization. Additionally, the identification of *cis*-acting elements, both experimentally and *in silico*, is much more difficult than that of the coding sequences in eukaryotic cells; therefore, the real extent of potential divergent overlaps is currently unknown.

***Convergently overlapping protein-coding genes***. The presence of overlaps between oppositely oriented genes has been described in diverse species, such as yeast (Peterson and Myers, 1993; Prescott and Proudfoot, 2002), *C. elegans* (Osato et al., 2007), and PRV (Szpara et al., 2011). Convergent genes can produce mRNAs with an overlap in their 3′-UTRs. However, very few convergent ORF–ORF overlapping has been detected, possibly because the determination of protein structure imposes a severe constraint on codon usage in the coding DNA strand, leaving limited variations of the evolution of protein-coding sequences on the other DNA strand. Convergent PRV genes are thought to be non-overlapping with their oppositely oriented partners. The only true convergently overlapping gene pair is the *ul30* and *ul31* (175-nucleotide total overlap with 75-nt overlap in the ORFs). Apparently, the transcriptional machineries of the opposing genes must collide at the overlapping region unless their expressions are temporally well separated. According to the Seesaw model, TI at the overlapping region plays a pivotal role in the temporal separation of the expression of overlapping genes. The underlying mechanism is based on either the dislocation of RNAP molecules, which results in the inhibition of the assembly of transcription pre-initiation complex and/or the premature termination of the transcription. This process provides an automatism through which a gene with a high expression rate suppresses the expression of the opposing gene(s) with lower activity. The alteration of the transcriptional direction in a locus is initiated by transcription factors (through a change in their composition and/or activity).

**FIGURE 4:**
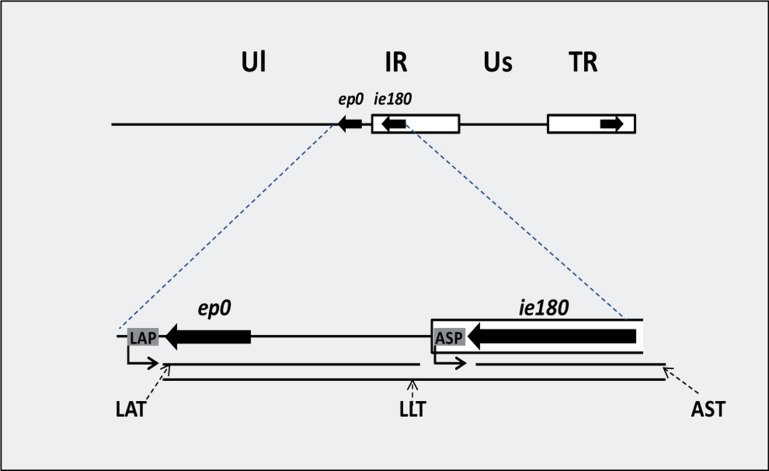
**Antisense transcripts regulating the two key transcriptional activators of pseudorabies virus**. The immediate early 180 (IE180) and early protein 0 (EP0) proteins are the major regulators of global gene expression of PRV. The latency-associated transcript (LAT), the antisense transcript (AST) and the long latency transcripts (LLT) are RNA molecules transcribed from the complementary DNA strands of these genes from their own promoters. The antisense transcripts are driven by LAP (latency-associated transcript promoter) and ASP (antisense promoter).

**FIGURE 5:**
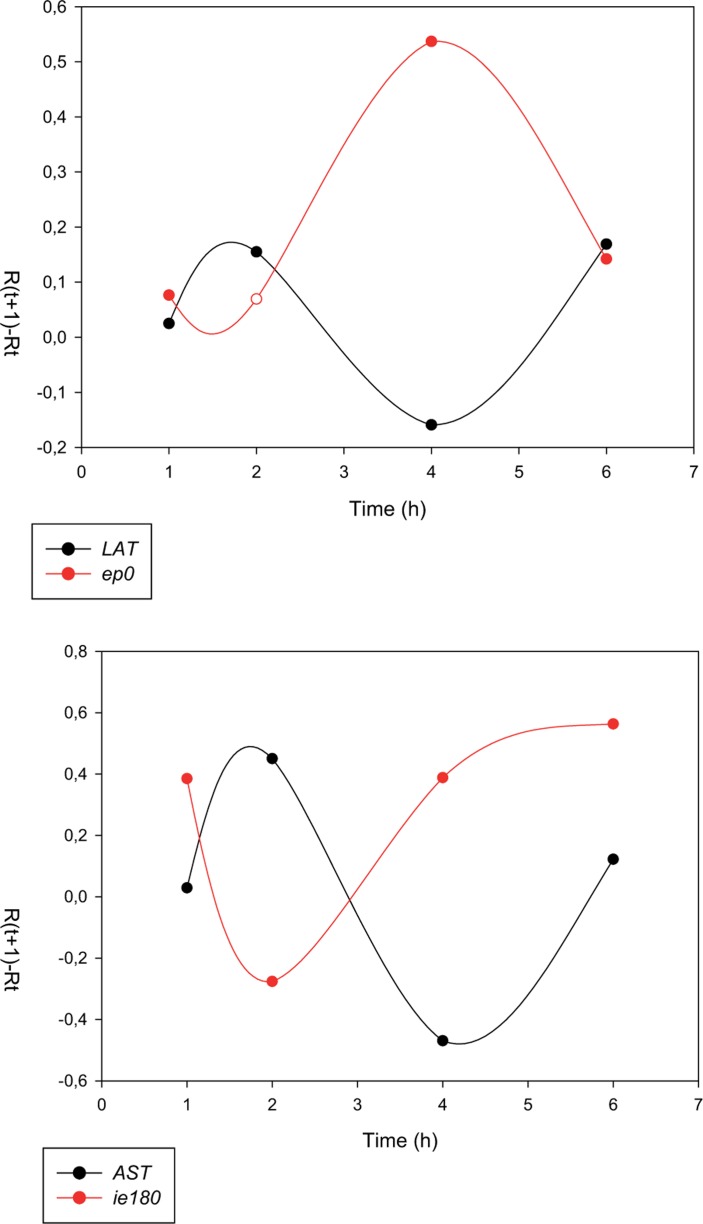
**Inverse expression pattern between the sense and antisense transcripts of pseudorabies virus**. The transcription rates (*R*_Δ_ values) of sense (mRNAs) and antisense RNAs exhibit reciprocal relationships [67]. That is, high transcriptional activity of mRNAs results in a decrease in the transcription rate of the antisense transcripts, and *vice versa*.

***Convergent overlap between a protein-coding gene and an RNA gene***. The overlaps between protein-coding genes and RNA genes have been described in several cellular organisms and viruses (Izant and Weintraub, 1985; Katayama et al., 2005; Lapidot and Pilpel, 2006). In this type of interaction, there is no constraint for protein coding in one of the overlapping partners, which allows the formation of long overlaps. There is increasing recognition that the antisense RNAs within the *Hox* cluster play an important role in the regulation of gene expression (Mainguy et al., 2007). The early protein 0 gene (*ep0*)-LAT and the *ie180*-AST transcript pairs serve as examples for this type of transcriptional overlap in PRV. Additionally, the LLT overlaps both *ep0* and *ie180* mRNAs (**Figure 4**). The HSV LAT has been shown to encode miRNAs (Umbach et al., 2008). It is still unknown whether the sole function of these antisense transcripts is being the precursor of miRNAs. In an earlier report, we investigated the alteration in the amount of sense (mRNA) and antisense transcripts during viral infection using real-time RT-PCR, and obtained an inverse relationship between them (Tombácz et al., 2009; **Figure 5**), indicating that the transcription rates of these molecules were interrelated. The LAT and AST transcripts of PRV are considered non-coding. Intriguingly, however, the complementary DNA strands contain long ORFs, which had led to the speculation of their potential in encoding antisense proteins. However, we showed that these ORFs were generated by the selective accumulation of G and C bases in the silent codon positions of these genes, thus eliminating stop codons from the fourth antisense reading frame on the complementary DNA strand (Boldogköi et al., 1995). We proposed that these antisense ORFs are the simple by-products of a selective GC-pressure, which, if they have any function, are involved in something else other than the generation of protein-coding sequences on the complementary DNA strand.

***Convergent read-through overlap between protein-coding genes***. Many genes in an organism are oriented in an opposite direction relative to each other. The herpesvirus genome appears to be organized in a modular fashion, each module (GM) being composed of two convergently oriented tandem gene clusters. The Seesaw model proposes the existence of a mechanism that allows an extensive transcriptional read-through across the oppositely oriented genes, which is the result of the inefficient termination of transcription at the terminal poly(A) signals of the nested gene clusters. This mechanism is supposedly under the control of the ICP27 protein, resulting in the generation of long transcripts containing sequences that are complementary to the minus DNA strand of a gene and antisense sequences transcribed from the plus strand of the opposing gene. While most of the genes within the tandem gene cluster belong to the same kinetic class, the convergent partners usually belong to different temporal classes (Tombácz et al., 2009). A special type of overlap occurs in the *ul15*, *ul16*, and *ul17* genes, where the two latter genes are embedded in the former one in a convergent orientation (**Figure 1**). The *ul15* gene has to produce a long transcript encompassing the other two genes, which are removed during mRNA processing by splicing. The short Us region appears to be differently organized than the long Ul region of the PRV genome: all the Us genes are tandemly oriented. However, the *us1* gene located on the inverted repeats is transcribed in an opposite direction (**Figure 1**). The Us genes are transcribed in an E/L kinetics (except *us3*, which is an E gene), while the *us1* (*icp22*) appears to be an L gene in PRV (Tombácz et al., 2009) and an IE gene in HSV (Prod’hon et al., 1996). It is predicted that a long transcript encompassing the entire Us region is generated from the *us1* gene as a result of transcriptional read-through from this gene. The “Winner takes all” principle can also be applied to the convergent read-through mechanisms. The long transcripts containing the antisense RNA and the mRNA of the opposite gene can be separated by cleavage at the poly(A) site of the mRNA or by removing the antisense transcript by RNA splicing.

#### The Extended Seesaw model

**FIGURE 6:**
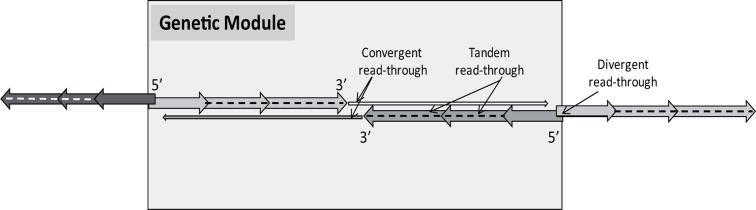
**A prototypical genetic module of the herpesviruses**. The PRV genome exhibits a modular design composed of tandemly overlapping genes, as opposed to another tandem gene cluster in the prototypical Genetic Modules. Many tandem genes are assumed to be transcriptionally overlapping in a parallel fashion (e.g., *ul42*, *ul43*, and *ul44*); however, no reports have been published on this to date.

The TIN hypothesis suggests the following scenario for controlling herpesvirus replication through the TINs. We considered prototypic GMs, which are composed of two opposing tandem gene clusters. Each tandem gene cluster is composed of genes with different kinetic subclasses and the two convergent gene clusters belong to different kinetic classes (**Figure 6**). However, real GMs can be built up somewhat differently: (1) the first step of the transcription cascade of PRV is the production of IE180 mRNA, which does not need *de novo* viral protein synthesis. The transcription of this transactivator is regulated, among others, by the RNA genes AST and LLT through a seesaw mechanism (**Figure 5**), and by its own protein product (auto-feedback; Tombácz et al., 2009).****(2) Next, several E genes are induced by the IE180 transactivator in association with specific cellular factors. At this stage of viral infection, the upstream E genes inhibit both their tandem gene partners through a waterfall mechanism and the convergent L genes through a seesaw mechanism. Furthermore, these upstream E genes also inhibit the expression of the divergently oriented genes belonging to different kinetic (sub)classes. The *ie180* and the *ep0* genes encode the two key transcriptional activators of PRV. Therefore, these genes and also their antisense partners require a complex regulation, which could explain why LAT, LLT, and AST are controlled by their own promoters. This is in contrast to the rest of the antisense RNAs, which, according to the Seesaw model, are controlled by the promoter of opposite genes and produced via a transcriptional read-through mechanism. (3) In the next step, the ICP27 protein and/or other factors recognize the poly(A) signal of the upstream E genes, thereby allowing the transcription of downstream E genes from their own promoters. (4) Subsequently, gene expression swaps from the E to L stage (capsizing the arms of the seesaw), in which the ICP27 protein may play a critical role by efficiently recognizing the poly(A) signals at the 3′ co-terminals leading to a decreased convergent read-through from the E genes. Furthermore, poly(A) signals at the 3′ co-terminal of the late genes may not be recognized by the RNAP molecule, causing a transcriptional read-through from the L genes toward the E genes. In other words, the poly(A) signals might be differentially recognized by ICP27 and/or other factors, thereby dictating a temporal order in the succession of the ON/OFF switching of gene expression. Other viral proteins, such as VHS and EP0, may also play roles in the kinetic shift, presumably through modulating the expression and/or activity of the IE180 transactivator (Tombácz et al., 2011, 2012). (5) Following the switch, the transcription of the L genes are initiated by the IE180 protein and other factors, which results in the inhibition of the E gene expression through the above-described read-through mechanism. In principle, the direction of the transcription can be changed more than once between convergent nested gene clusters in a hypothetical configuration of E1L1–L2E2 in the following temporal order: (step 1) E1 gene inhibits both the tandem L1 gene and the convergent L2 and E2 genes; (step 2) E2 inhibits all of the other three genes; (step 3) L1 blocks L2 and E2 genes; (step 4) L2 inhibits E1 and L1 (E1 and E2 genes are silent at steps 3 and 4 due to the absence of transcriptional initiation). The cascade-like events of viral gene expression is explained in terms of the mechanisms based on the combination of parallel and antiparallel collisions of RNAP molecules at the overlapping regions, hence, the name “Extended Seesaw model.” Adjacent GMs may influence one another’s transcription via transcriptional read-through extending beyond the boundaries separating two GMs. The long RNA molecules synthesized by the above processes may be difficult to detect by “size-sensitive” techniques, such as Northern blot analysis, because they may be randomly terminated and thus, produce smears on the gel.

#### The Promoter Competition model

Bidirectional promoters can also function in the regulation of the temporal orderliness of gene expression. Two of the divergent gene pairs of PRV (*ul20*–*ul21* and *ul29*–*ul30* genes) overlap at their TATA boxes. In our earlier publication, we categorized the *ul20* gene as E/L, and the other three genes as E genes. It is assumed that these genes are expressed in a slightly different kinetics, which is, at least partly, regulated by competition between overlapping promoters for the transcriptional initiation factors. The mechanisms influencing the preferential binding of transcription factors to the two regions of these promoters are currently unknown.

### TINs IN CELLULAR ORGANISMS

The operating mechanism of TINs has been demonstrated through use of a herpesvirus model. The question may be raised of whether the same or similar principles can be applied to cellular organisms too. The structure and operation of genes of metazoan organisms are more complex than those of viral genes, due to the presence of introns, and the extended leader and trailer sequences, the need for a multitude of *cis*-regulatory sequences and transcription factors for the proper expressions, the essential roles of epigenetic modifications, and the expansive intergenic regions. Although viruses are intracellular parasites which exploit the biochemical and genetic apparatuses of the cell, it is theoretically possible that they utilize essentially different TIN-based mechanisms for the control of their gene expressions. The differences and similarities between these biological systems are explored below.

#### Parallel read-through by the tandem genes

The tandem organization of genes is common throughout the genome in all species (Pan and Zhang, 2008). For example, the vertebrate *Hox* genes are all positioned in a tandem orientation relative to one other (**Figure 2**). An extensive transcriptional read-through between the *Hox* genes has been reported in various species (Simeone et al., 1988; Shiga et al., 2006), indicating the importance of this mechanism in diverse evolutionary lineages. Furthermore, it has been demonstrated that, besides the “regular” transcriptional start site (TSS), many yeast genes contain an additional upstream TSS, which controls the generation of short CUT molecules transcribed in the same direction as those of mRNAs, which overlap the promoter and 5′-UTRs of the mRNAs (Neil et al., 2009). It has been shown that the mutation of the TATAA box of the yeast *TPI1* gene affects the transcription of the mRNA, but not that of the CUT, which indicates the existence of two independent pre-initiation complexes on the promoter (Scott and Baker, 1993). Furthermore, the same study revealed a reverse correlation between the expressions of CUTs and the mRNAs of downstream glycolytic genes, which may reflect the operation of a mechanism similar to that described in the herpesviruses in the Waterfall model. The difference between these two systems is that in yeasts the upstream transcripts are not read-through products of upstream protein-coding genes, but are from RNA genes producing CUTs. Additionally, global run-on sequencing (Gro-seq) analysis has revealed that human RNAP extends more than 10 kb pairs on average beyond the poly(A) site of genes before termination (Core et al., 2008), and may interact with the transcription of closely positioned tandem genes, if they overlap.

#### Convergent gene overlaps

The estimated extent of the overlapping transcripts varies between the different organisms: 29% in mouse (34.9% in the protein-coding regions; Katayama et al., 2005), 5% in rat (Sun et al., 2006), 22% in human (Chen et al., 2004), 15% in fruit fly (Misra et al., 2002), and 9% in *Arabidopsis* (Wang et al., 2005). Sun et al. (2006) modified the data for mouse (12%) and fruit fly (17%). On the other hand, Cheng et al. (2005), using RACE/tiling arrays, found that 61% of all human transcribed regions have a counterpart on the opposite strand. These data demand further clarification, in view of the significant variations in the results obtained for the same species in different laboratories. Furthermore, it is difficult to accept that the mouse and rat differ to such a great extent as indicated above. The discrepancies in the data could be a consequence of the different techniques used by the different research groups. CUTs generated from a bidirectional promoter can overlap and thereby, interfere with a gene oriented in divergent fashion relative to a gene having a bidirectional promoter.

***Convergent and divergent overlaps between protein-coding genes***. The *ul30 and ul31* genes are the only true convergently overlapping protein-coding gene pair of PRV. Convergent overlaps between oppositely oriented genes, as in the yeast RHO1/MRP2 (Peterson and Myers, 1993) and GAL7/GAL10 gene pairs (Prescott and Proudfoot, 2002), appear to be rare in cellular organisms too. The reason for this could be that this type of interaction imposes a severe constraint on the expression of genes. Similarly, divergent gene overlaps are probably also rare, since gene expression is regulated by a multitude of other mechanisms in complex genomes.

**FIGURE 7:**
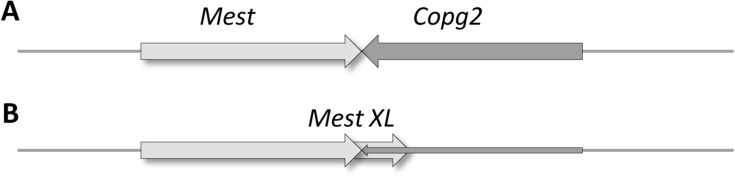
**Alternative polyadenylation leads to TIN between opposing genes**. The *Mest* and *Copg2* genes are positioned in a convergent orientation relative to each other on the mouse DNA. Transcription of the *Mest* gene is terminated “normally,” without overlapping with the *Copg2* gene in embryonic liver cells **(A)**, whereas *Mest* transcription is continued within the *Copg2* gene in neurons, thereby reducing the expression of this gene **(B)**.

***Convergent read-trough overlaps between protein-coding genes***. The idea of convergent transcriptional read-trough assumes the existence of a main transcriptional termination/poly(A) signal, which is occasionally overlooked by the RNAP enzyme. Eukaryotic genes do not necessarily form convergent read-through overlaps to achieve a similar regulatory effect. Instead, they can also overlap as a result of alternative polyadenylation, which is recognized as a major post-transcriptional regulatory mechanism in eukaryotes. Differential polyadenylation depends on a variety of factors, including cell type (such as the *Mest* and *Cop* genes of the mouse; MacIsaac et al., 2011; **Figure 7**), embryonic stage, environment and disease (Audic and Claverie, 1997). The 3′-UTRs are largely unannotated, and the extent of alternative polyadenylation in the various genomes is therefore still unknown. Through use of a novel genomic 3′RACE technique, Mangone et al. (2010) detected the presence of at least one 3′-UTR isoform for every protein-coding gene of *C. elegans*, which were differentially expressed during development. The extents of the various overlaps produced by alternative polyadenylation are currently unknown.

***Convergent overlaps between a protein-coding gene and an RNA gene***. The *Hox* gene cluster is one of the best-known groups of genes, but it is still not available a comprehensive knowledge relating to the transcription from these DNA regions. It was recently estimated that the unannotated transcripts can account for up to 60% of the total transcriptional output of a cluster (Mainguy et al., 2007). The generation of antisense transcripts has been reported in *Drosophila Hox* genes, which act in both *trans* (Rinn et al., 2007) and in *cis;* this latter represents 14 antisense transcripts overlapping the *Hox* genes (Mainguy et al., 2007). Antisense CUTs generated from a bidirectional promoter can, in principle, convergently overlap with the 3′-UTR of an upstream tandem gene over a certain length. This potential mechanism could provide a feedback from the downstream gene toward the upstream gene, which would provide a seesaw-like mechanism. An overall antisense transcription has been described in the mammalian genome. A perturbation of antisense transcription has been shown to affect the expression of mRNAs, which suggests that the antisense transcripts and/or the process generating them are functional (Katayama et al., 2005).

***Convergent overlaps between RNA genes***. A classical example of the interaction between two lnc-RNAs involves the *Xist* and *Tsix* RNA genes. Xist is the major factor of X chromosome inactivation, while Tsix is a *cis*-acting repressor of Xist. A close correlation has been established between Xist downregulation and Tsix upregulation during X inactivation (Rougeulle and Avner, 2004). A genome-wide occurrence of overlaps between the RNA genes has been reported in the mammalian genome (Katayama et al., 2005), which provides a great number of possibilities for the operation of TINs. It is reasonable to assume that the RNA genes, and especially those encoding lnc-RNAs, are regulated by the interaction of the transcriptional machineries according to the Waterfall, Seesaw, or Promoter Competition models.

***Bidirectional promoters***. A large proportion of eukaryotic promoters are intrinsically bidirectional (Wei et al., 2011), though the explanation of this is not yet known. When a bidirectional promoter controls the transcription of two divergent protein-coding genes, the purpose could be to provide either a synchronized expression or, in contrast, an inversely synchronized expression, as predicted by the Promoter Competition model. In most cases, however, bidirectional promoters generate the short, unstable transcripts called CUTs in the direction opposite to the genes. If a CUT does not overlap with a gene, it may result as a by-product of promoter competition (see above for other possible mechanisms). If a CUT overlaps with an upstream gene, then, depending on the relative orientation of this gene relative to the downstream gene, the interaction between the transcriptional machineries of the upstream gene and the CUT can be unidirectional (tandem orientation) or bidirectional (convergent orientation), as explained by the Waterfall model or the Seesaw model, respectively. Indeed, serial analysis of gene expression (SAGE) has shown that the 3′ ends of CUTs can be located in close proximity to the ORFs in both sense and antisense configurations (Neil et al., 2009).

A major difference between viruses and cellular organisms is that the interacting partner of a protein-coding gene in a cellular organism, and especially in a eukaryote, appears to be more frequently an RNA gene, rather than another protein-coding gene as in a virus. The reason for this could be that viruses contain extremely compact and efficient genomes, since they have to utilize every detail of the DNA. In PRV, only the two most important transactivator genes (*ie180* and *ep0*) are regulated by antisense transcription from RNA genes (LAT and ASP). Additionally, at the moment, it is not known whether CUTs are also expressed in viruses.

### THE FUNCTION OF TINs

The TIN hypothesis claims that TIN-based mechanisms are not only curiosities with peripheral significance, but control the operation of a wide range of gene clusters throughout the genome. The temporal control of gene expression is assumed to be especially important in a variety of mechanisms, including virus life cycle, embryogenesis, cell differentiation, tissue regeneration, cell signaling pathways, cellular stress, operation of gene networks, and metabolic pathways, among others. TINs can also participate in simple regulatory mechanisms requiring mutually exclusive gene expression of neighboring genes. Besides the temporal regulation, TINs are assumed to be involved in the spatial control of transcription in multicellular organism, which includes the regulation of tissue-specific gene expression.

### THE POTENTIAL FUNCTIONS OF READ-THROUGH TRANSCRIPTS

Antisense transcripts and polycistronic RNAs are the by-products of the operation of TINs. However, this does not inevitably mean that they are not utilized at other levels of genetic regulation or in other processes. Antisense RNAs can serve as miRNA precursors (Umbach et al., 2008), as well as translational regulators, by binding the complementary mRNA strand, thereby forming double-stranded RNAs. Indeed, the presence of double-stranded transcripts has been detected in cells infected with HSV (Jacquemont and Roizman, 1975), a mouse polyoma virus (overlapping poly(A) signals; Osato et al., 2007) and in *Drosophila* (Lai, 2002; Lewis et al., 2005). One might speculate about the function of long double-stranded viral transcripts in herpesvirus-infected cells. The mRNA/antisense RNA duplexes normally evoke the interferon-induced apoptotic pathway in mammalian cells. However, herpesviruses have developed several anti-apoptotic mechanisms as a defense strategy (Li and Ramchandran, 2010). Even though double-stranded transcripts are tolerated by infected cells, the viral life cycle is too rapid for the completion of RNA interference. Alternatively, antisense transcripts may act as epigenetic regulators in processes such as the methylation of promoters and the conversion of the chromosome structure (Wutz et al., 1997; Reik and Walter, 2001; Tufarelli et al., 2003). Additionally, downstream genes of polycistronic RNAs can be translated through any of the abovementioned mechanisms.

## CONCLUSION

Protein and RNA molecules are needed in optimal amounts in order to fulfill their biological functions; therefore, the regulation of their availability on a spatiotemporal scale is a critical point for the normal operation of cells and the organisms themselves. The TIN hypothesis suggests the genome-wide existence of GMs coordinating the expression of its genes through the interaction between their transcriptional machineries. The interplay among genes within and between GMs forms a TI network, which represents a novel layer of gene regulatory control in various forms of life. The TIN hypothesis suggests that the role of a TIN includes controlling the ON/OFF switching of gene expression in a temporally successive manner and maintaining the ON or OFF state for a given period of time within a cluster of functionally related genes through disrupting each other’s transcription at the initiation and/or elongation phase. The transcription machineries clash with each other at overlapping regions in the collision-based (Waterfall and Seesaw) models and compete with each other in the promoter-competition-based model. In the collision-based models, the genes with higher initial expression rates and/or transcriptional read-through efficiencies suppress the transcription of genes with lower initial expression and/or efficiency, thereby further reducing the transcription of the latter genes close to the zero level. The Waterfall model predicts a one-way inhibitory effect (from upstream to downstream genes) among tandem genes, while the Seesaw model envisages a reciprocal downregulatory effect on the transcription of convergently oriented genes. The latter system results in an inverse synchronization of transcription between convergent genes through a self-adjusting automatism, thereby greatly simplifying the coordination of gene expression. The Extended Seesaw model proposes a complex regulatory pattern between genes forming both tandem and convergent clusters in a genomic locus. In the latter case, the direction of transcription can be repeatedly altered. In this study, we focused on the operation of TINs in alpha-herpesviruses. The question is whether the large-scale organization of the genomes of cellular organisms resembles that of the herpesviruses. The number of true convergent overlaps between protein-coding genes is low in the genomes of various cellular organisms, which appears to imply a peripheral significance of transcriptional collision in controlling genome-wide transcription. However, convergent orientation of gene clusters is common in these organisms, which potentially allow transcriptional read-through and alternative transcriptional termination mechanisms. Moreover, the intrinsic bidirectionality of the eukaryotic promoters provides an additional means for neighboring genes to exert a mutual effect on each other’s expression.

## Conflict of Interest Statement

The author declares that the research was conducted in the absence of any commercial or financial relationships that could be construed as a potential conflict of interest.

## Abbreviations

AST: antisense transcript
CUT: cryptic unstable transcript
E: early
E/L: early/late
GM: genetic module
HSV: herpes simplex virus
IE: immediate-early
IRES: internal ribosome entry site
L: late
LAT: latency-associated transcript
LLT: long latency transcript
Lnc-RNA: long non-coding RNA
miRNA: micro RNA
NAT: natural antisense transcript
ORF: open reading frame
Pi: post-infection
PRV: pseudorabies virus
RNAP: RNA polymerase II
TI: transcriptional interference
TIN: transcriptional interference network
Ul: unique long
Us: unique short
UTR: untranslated region
